# Supplementary material for: DNA Polymerase Delta Exhibits Altered Catalytic Properties on Lysine Acetylation
Source: Genes (Basel). 2023 Mar 23;14(4):774. doi: 10.3390/genes14040774 (PMC10137900; doi:10.3390/genes14040774)
Supplement: Supplementary file 1 [file genes-14-00774-s001.zip › genes-2186668-supplementary.pdf]

Article

DNA Polymerase Delta Exhibits Altered Catalytic Properties on Lysine Acetylation

Catherine Njeri[1], Sharon Pepenella[3], Robert A. Bambara[3] and Lata Balakrishnan [1,2,*]

Supplementary Figure S1A

In Vitro Acetylated Pol δ

DPOD1_HUMAN (100%), 123,633.5 Da
DNA polymerase delta catalytic subunit OS=Homo sapiens GN=POLD1 PE=1 SV=2
73 exclusive unique peptides, 114 exclusive unique spectra, 197 total spectra, 880/1107 amino acids (79% coverage)

1 MDGKRRPGPG PGVPPKRARG GLWDDDDAPR PSQFEEDLAL MEEMEAEHRL QEQEEEELQS VLEGVADGQV PPSAIDPRWL RPTPPALDPQ
91 TEPLIFQQLE IDHYVGPAQP VPGGPPPSRG SVPVLRAFGV TDEGFSVCCH IHGFAPYFYT PAPPGFGPEH MGDLQRELNL AISRDSRGGR
181 ELTGPAVLAV ELCSRESMFG YHGHGPSPFL RITVALPRLV APARRLLEQG IRVAGLGTPS FAPYEANVDF EIRFMVDTDI VGCNWLELPA
271 GKYALRLKEK ATQCQLEADV LWSDVVSHPP EGPWQRIAPL RVLSFDIECA GRKGIFPEPE RDPVIQICSL GLRWGEPEPF LRLALTLRPC
361 APILGAKVQS YEKEEDLLQA WSTFIRIMDP DVITGYNIQN FDLPYLISRA QTLKVQTFPF LGRVAGLCSN IRDSSFQSKQ TGRRDTKVVS
451 MVGRVQMDML QVLLREYKLR SYTLNAVSFH FLGEQKEDVQ HSIITDLQNG NDQTRRRLAV YCLKDAYLPL RLLERLMVLV NAVEMARVTG
541 VPLSYLLSRG QQVKVVSQLL RQAMHEGLLM PVVKSEGGED YTGATVIEPL KGYYDVPIAT LDFSSLYPSI MMAHNLCYTT LLRPGTAQKL
631 GLTEDQFIRT PTGDEFVKTS VRKGLLPQIL ENLLSARKRA KAELAKETDP LRRQVLDGRQ LALKVSANSV YGFTGAQVGK LPCLEISQSV
721 TGFGRQMIEK TKQLVESKYT VENGYSTSAK VVYGDTDSVM CRFGVSSVAE AMALGREAAD WVSGHFPSPI RLEFEKVYFP YLLISKKRYA
811 GLLFSSRPDA HDRMDCKGLE AVRRDNCPLV ANLVTASLRR LLIDRDPEGA VAHAQDVISD LLCNRIDISQ LVITKELTRA ASDYAGKQAH
901 VELAERMRKR DPGSAPSLGD RVPYVIISAA KGVAAYMKSE DPLFVLEHSL PIDTQYYLEQ QLAKPLLRIF EPILGEGRAE AVLLRGDHTR
991 CKTVLTGKVG GLLAFAKRRN CCIGCRTVLS HQGAVCEFCQ PRESELYQKE VSHLNALEER FSRLWTQCQR CQGSLHEDVI CTSRDCPIFY
1081 MRKKVRKDLE DQEQLLRRFG PPGPEAW

DPOD4_HUMAN (100%), 12,433.3 Da
DNA polymerase delta subunit 4 OS=Homo sapiens GN=POLD4 PE=1 SV=1
9 exclusive unique peptides, 11 exclusive unique spectra, 22 total spectra, 87/107 amino acids (81% coverage)

1 MGRKRLITDS YPVVKRREGP AGHSKGELAP ELGEEPQPRD EEEAELELLR QFDLAWQYGP CTGITRLQRW CRAKQMGLEP PPEVWQVLKT
91 HPGDPRFQCS LWHLYPL

Supplementary Figure S1B

Immunoprecipitated Pol δ from HEK293T Cells

DPOD1_HUMAN (100%), 123,633.5 Da
DNA polymerase delta catalytic subunit OS=Homo sapiens GN=POLD1 PE=1 SV=2
42 exclusive unique peptides, 50 exclusive unique spectra, 138 total spectra, 634/1107 amino acids (57% coverage)

```
1    MDGKRRPGPG PGVPPKRARG GLWDDDDAPR PSQFEEDLAL MEEMEAEHRL QEQEEEELQS VLEGVADGQV PPSAIDPRWL RPTPPALDPQ
91   TEPLIFQQLE IDHYVGPAQP VPGGPPPSRG SVPVLRAFGV TDEGFSVCCH IHGFAPYFYT PAPPGFGPEH MGDLQRELNL AISRDSRGGR
181  ELTGPAVLAV ELCSRESMFG YHGHGPSPFL RITVALPRLV APARRLLEQG IRVAGLGTPS FAPYEANVDF EIRFMVDTDI VGCNWLELPA
271  GKYALRLKEK ATQCQLEADV LWSDVVSHPP EGPWQRIAPL RVLSFDIECA GRKGIFPEPE RDPVIQICSL GLRWGEPEPF LRLALTLRPC
361  APILGAKVQS YEKEEDLLQA WSTFIRIMDP DVITGYNIQN FDLPYLISRA QTLKVQTFPF LGRVAGLCSN IRDSSFQSKQ TGRRDTKVVS
451  MVGRVQMDML QVLLREYKLR SYTLNAVSFH FLGEQKEDVQ HSIITDLQNG NDQTRRRLAV YCLKDAYLPL RLLERLMVLV NAVEMARVTG
541  VPLSYLLSRG QQVKVVSQLL RQAMHEGLLM PVVKSEGGED YTGATVIEPL KGYYDVPIAT LDFSSLYPSI MMAHNLCYTT LLRPGTAQKL
631  GLTEDQFIRT PTGDEFVKTS VRKGLLPQIL ENLLSARKRA KAELAKETDP LRRQVLDGRQ LALKVSANSV YGFTGAQVGK LPCLEISQSV
721  TGFGRQMIEK TKQLVESKYT VENGYSTSAK VVYGDTDSVM CRFGVSSVAE AMALGREAAD WVSGHFPSPI RLEFEKVYFP YLLISKKRYA
811  GLLFSSRPDA HDRMDCKGLE AVRRDNCPLV ANLVTASLRR LLIDRDPEGA VAHAQDVISD LLCNRIDISQ LVITKELTRA ASDYAGKQAH
901  VELAERMRKR DPGSAPSLGD RVPYVIISAA KGVAAYMKSE DPLFVLEHSL PIDTQYYLEQ QLAKPLLRIF EPILGEGRAE AVLLRGDHTR
991  CKTVLTGKVG GLLAFAKRRN CCIGCRTVLS HQGAVCEFCQ PRESELYQKE VSHLNALEER FSRLWTQCQR CQGSLHEDVI CTSRDCPIFY
1081 MRKKVRKDLE DQEQLLRRFG PPGPEAW
```

DPOD4_HUMAN (100%), 12,433.3 Da
DNA polymerase delta subunit 4 OS=Homo sapiens GN=POLD4 PE=1 SV=1
10 exclusive unique peptides, 13 exclusive unique spectra, 24 total spectra, 87/107 amino acids (81% coverage)

```
1    MGRKRLITDS YPVVKRREGP AGHSKGELAP ELGEEPQPRD EEEAELELLR QFDLAWQYGP CTGITRLQRW CRAKQMGLEP PPEVWQVLKT
91   HPGDPRFQCS LWHLYPL
```

Supplementary Figure S1: Scaffold image of amino acid sequence of Pol δ
Peptide sequences detected by mass spectrometric analysis are highlighted in yellow. Lysine residues detected have a +42 dalton change were identified as acetylated residues and have been highlighted in green. Oxidized methionine residues served as dynamic modification controls (also highlighted in green). Supplementary Figure 1A displays the sequence obtained from in vitro acetylated Pol d and Figure 1B displays the sequence obtained from endogenous polymerase that was immunoprecipitated from HEK293T cells. Coverage obtained is also indicated above the sequence.

Supplementary Figure S2

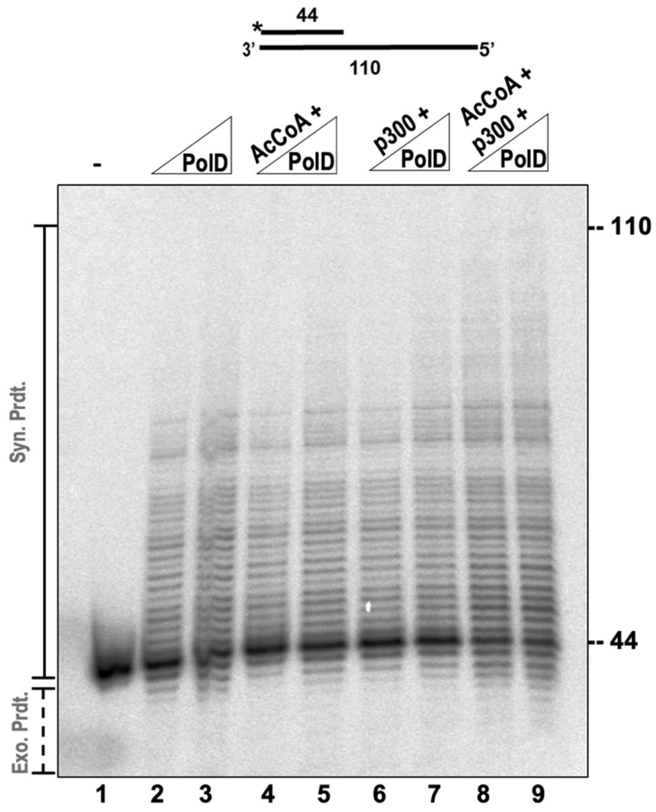

Supplementary Figure S2: Synthesis Activity of Pol Delta

The synthesis activity of pol δ was assayed using the 5nM substrate (U_1:T_1) in the presence of varying concentration (20, 50 nM) of either pol pol δ, pol δ + AcCoA, pol δ + p300 (HAT domain) and acetylated pol δ (pol δ+AcCoA+p300) as described in Materials and Methods. The substrate is depicted above the gel image and the asterisk denotes the location of the radiolabel. Exonuclease products (Exo.Prdt.), and synthesis products (Syn, Prdt.) are indicated adjacent to the gel image. Nucleotide sizes are also indicated.
